# Decomposition Dynamics of a New Noble-Gas Compound

**DOI:** 10.3390/molecules30224398

**Published:** 2025-11-14

**Authors:** Arik Cohen, Robert Benny Gerber

**Affiliations:** 1 The Fritz Haber Center for Molecular Dynamics, Institute of Chemistry, The Hebrew University, Jerusalem 91904, Israel; aricor99@gmail.com; 2 Department of Chemistry, University of California, Irvine, CA 92697, USA

**Keywords:** HXeNH_2_ compound, noble-gas hydrides, dissociation pathway, cryogenic temperatures, ab initio, Ab Initio Molecular Dynamics (AIMD), partial charges rearrangement

## Abstract

This paper deals with the stability and decomposition of a recently predicted noble-gas compound, HXeNH_2_. Despite natural progress and interest in noble-gas hydrides, little is known of their decomposition reaction. In this study, the dissociation reaction is explored by ab initio calculations and by Ab Initio Molecular Dynamics (AIMD) simulations. The results could be important for the future experimental search of the compound. It is found that the main decomposition channel is HXeNH_2_ → H+Xe+NH_2_. A key step in the reaction is found to be a rearrangement of the partial charges of the atoms involved. The results could be of significance also for reactions of other compounds with Xe-N chemical bond and other noble-gas hydrides.

## 1. Introduction

A renaissance in the field of noble-gas chemistry occurred about three decades ago, with the discovery of the noble-gas (Ng) hydrides family of molecules [1]. These interesting compounds, which were discovered by Räsänen and coworkers [2], share a common structure formula of HNgY (where Ng = noble-gas and Y = an electronegative atom/group) and are characterized by relatively weak bonding and large dipole moments. The latter arises from the common ionic structure of (H-Xe)^+^Y^−^ [3]. These properties enhance the effects of their surrounding on them, their complexation, and their reactions with other species [4,5,6].

Notable representatives of this family include molecules such as HArF [7], HKrCl [2,8], HXeCCH [9], HKrCCH [10], and HXeOH [11,12]. Another distinguished group of researchers who prepared such molecules was that of Feldman and coworkers [12,13,14]. It should be noted that many predicted Ng hydride molecules still await their experimental realization [15,16].

Over the years, many types of chemical bonds of noble-gas elements with other atoms have been discovered. Among them is the important Xe-N bond, which dates back to the early 1970s, with the spectroscopical observation of FXeN(SO_2_F)_2_ [17]. Since then, the Xe-N chemistry has considerably expanded to include many compounds [18,19,20,21,22]. The important Xe-N bond found its place also in the “noble-gas renaissance era”, in compounds prepared by the Räsänen group, such as HXeNC, HXeNCO, FXeNC, and ClXeNC [23,24,25,26].

The structure, properties, and energetics of predicted and experimentally found noble-gas hydrides have been addressed along the years. Much less so is their stability at various temperatures and their decomposition dynamics and dissociation mechanism via AIMD. Studies of the latter include those by Tsivion and Gerber [27,28].

Here, we report on the decomposition dynamics at various temperatures and the dissociation mechanism of the recently predicted HXeNH_2_ molecule [29], a new prediction of noble-gas hydride type of molecule containing a Xe-N bond. In addition, we shed light on the role played by modifications in the electronic structure of the molecule versus structural changes that lead to its decomposition. Therefore, this study may help in the experimental discovery of HXeNH_2_ and may also bear significance for reactions of other compounds with Xe-N chemical bond and noble-gas hydrides.

It should be noted that recently, the theoretical prediction of the interesting ionic species NXeH_4_^+^ and HXeNH_3_^+^ were reported. However, although related, the neutral HXeNH_2_ in focus differs from the ionic species [30].

In addition, we propose that the HXeNH_2_ may be relevant to the known and long-standing puzzle of the “missing” Xe phenomenon in the Earth’s atmosphere [31,32,33]. Evidence indicates that Xe and NH_3_ species interact to give a stable complex at high pressure, relevant to planetary interiors [34,35]. Furthermore, spectroscopic and X-ray diffraction experiments on Xe-N_2_ mixtures at high pressure indicate the formation of species with strong Xe-N bonds [36]. Sanloup and coworkers demonstrated the reaction of Xe and ice at very high pressures for the closely related case of Xe-O [37]. Though we do not present any evidence for our proposal, we wish to raise here a plausible explanation for this phenomenon. If indeed some part of the “missing” Xe is due to the presence of currently unobserved HNgY molecules, then HXeNH_2_ may be involved due to the relatively high abundance of ammonia. Moreover, the HXeNH_2_ molecule is predicted to exist in non-pressurized environments, and there is evidence that HNgY compounds become more stable when pressurized. Thus, this suggests that HXeNH_2_ and others alike may be found beyond high-pressure planetary interiors as well.

The structure of the paper is as follows: the Section 2 begins with a brief presentation of the molecule and its properties. This is followed by the main subsection devoted to the presentation and discussion of the molecule’s decomposition dynamics at various temperatures and its dissociation mechanism. Next, a section describing the methodology and the computational methods that were used is outlined. And finally, the conclusions of this study are presented.

## 2. Results and Discussion

### 2.1. HXeNH_2_: Structure and Properties

**Figure 1:**
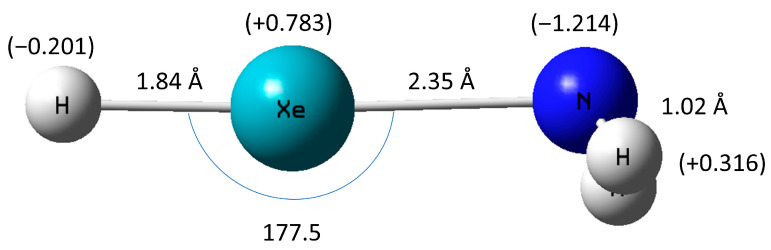
Optimized structure of HXeNH_2_. Distances (in Angstrom), angles (in degrees), and NBO partial atomic charges in parentheses (see also optimized coordinates in Table A3).

The calculated geometry of the predicted HXeNH_2_ at the CCSD(T) level of theory is shown in Figure 1.

The optimized HXeNH_2_ geometry exhibits bond lengths of 1.84 Å, 2.35 Å, and 1.02 Å for the H-Xe, Xe-N, and N-H bonds, respectively. Interestingly, at the CCSD(T) level of theory, the H-Xe and Xe-N bonds are longer in correspondence by only 0.16 Å and 0.20 Å than their counterparts in the rather similar HXeF molecule. In addition, the H-Xe bond is longer by 0.13 Å and shorter by 0.33 Å than the H-Xe and Xe-Cl bonds in the experimentally known HXeCl compound. Furthermore, not surprisingly, MP2 and CCSD(T) agree very closely on the optimized structure of HXeNH_2_. At the MP2 level of theory, the H-Xe bond and the Xe-N bond are shorter by 0.05 Å and 0.04 Å, compared with CCSD(T), respectively.

Similarly to other members of the noble-gas hydrides, the H-Xe-N angle is almost linear, at 177.5 degrees [24,38].

HXeNH_2_ shows a rather typical partial charge distribution among its atoms [39]. The hydrogen atom, which is bonded with xenon, is negatively charged (−0.201), while the Xe atom carries most of the positive charge in the molecule, +0.783. The NH_2_ subunit is altogether negatively charged, with a negative charge of −1.214 on the nitrogen and a positive charge of +0.316 on each of the hydrogen atoms bonded with it. This gives a negative net charge of −0.582 on the NH_2_ subunit.

The H-Xe vibrational mode is of special significance as it serves as a fingerprint for the experimental identification of the noble-gas compound in question [40]. In order to estimate the anharmonic vibrational frequency of the H-Xe bond in HXeNH_2_, the ratio of the experimental anharmonic to the harmonic vibrational frequency of that bond in the similar HXeCl molecule was calculated. The resulting factor of about 0.92 was then applied on the harmonic frequency of the H-Xe bond in HXeNH_2_ (1580.9 cm^−1^ and 1318.5 cm^−1^). This gives an estimated anharmonic frequency of 1213.0 cm^−1^ and 1454.4 cm^−1^ for the H-Xe bond in HXeNH_2_ (for all other HXeNH_2_ harmonic frequencies, see Table A1 in Appendix A). It should be noted that the anharmonic estimated frequencies for HXeNH_2_ correspond to a covalent H-Xe bond that should be detected experimentally.

**Figure 2:**
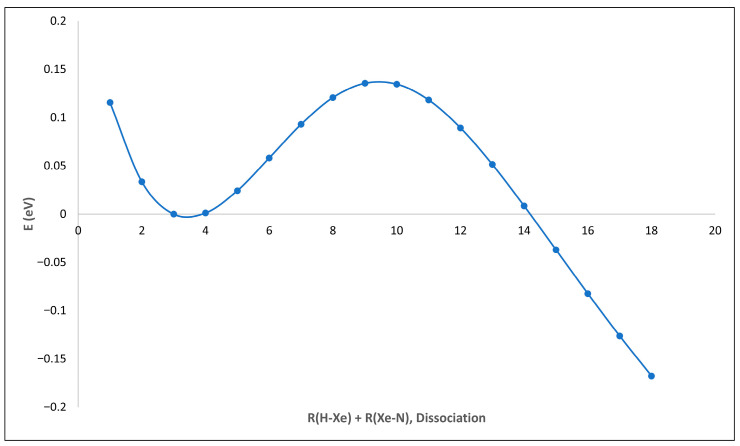
The decomposition pathway of HXeNH_2_ into H+Xe+NH_2_, calculated with MRCI(16,12).

Like all other members of the noble-gas hydrides, HXeNH_2_ is metastable [39,41], residing ~4.82 eV above the global minimum of the system, Xe+NH_3_. The barrier protecting the decomposition of HNgY into Ng+HY is usually much higher than that which hinders the dissociation of noble-gas hydrides into H+Ng+Y. Furthermore, it is believed that the former decomposition route is hindered in the matrix [39]. Therefore, the focus here shall be on the latter, namely the dissociation of HXeNH_2_ into H+Xe+NH_2_. The energy difference between HXeNH_2_ and its products, H+Xe+NH_2_, was calculated via both CCSD(T) and MP2 methods. The calculations give an energy difference of 0.39 eV and 0.40 eV for the CCSD(T) and the MP2 method, respectively. In addition, the decomposition pathway calculated via MRCI(16,12) gives a protecting barrier of about 0.13 eV (see Figure 2 and the Section 3).

This seemingly relatively low barrier is the estimated protecting barrier for this decomposition channel in vacuum. However, the noble-gas hydrides are usually prepared in a noble-gas matrix at cryogenic temperatures (T ≤ 10 K). Therefore, such a barrier should be sufficient for the stabilization and detection of the molecule at these extreme conditions. It should be noted that for the experimentally found HArF molecule, a similar barrier of 0.18 eV was calculated for this dissociation pathway using the same method applied here [7,42].

### 2.2. HXeNH_2_: Decomposition Dynamics at Cryogenic Temperatures

**Figure 3:**
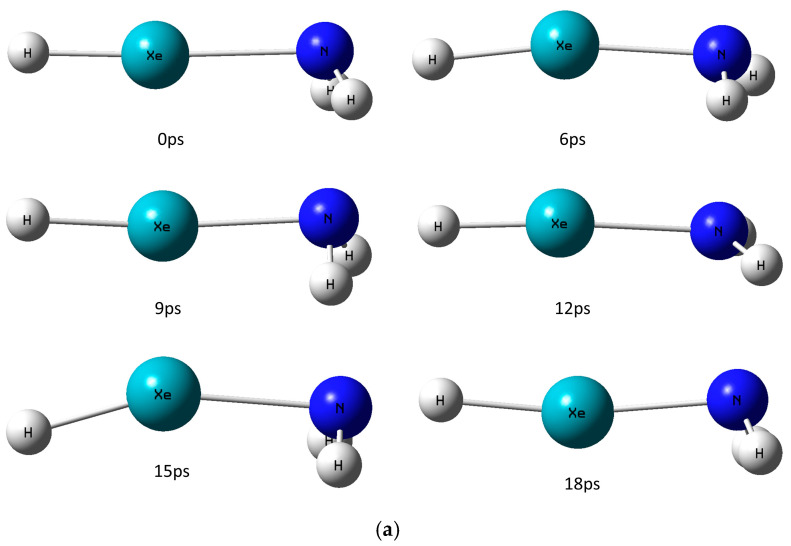

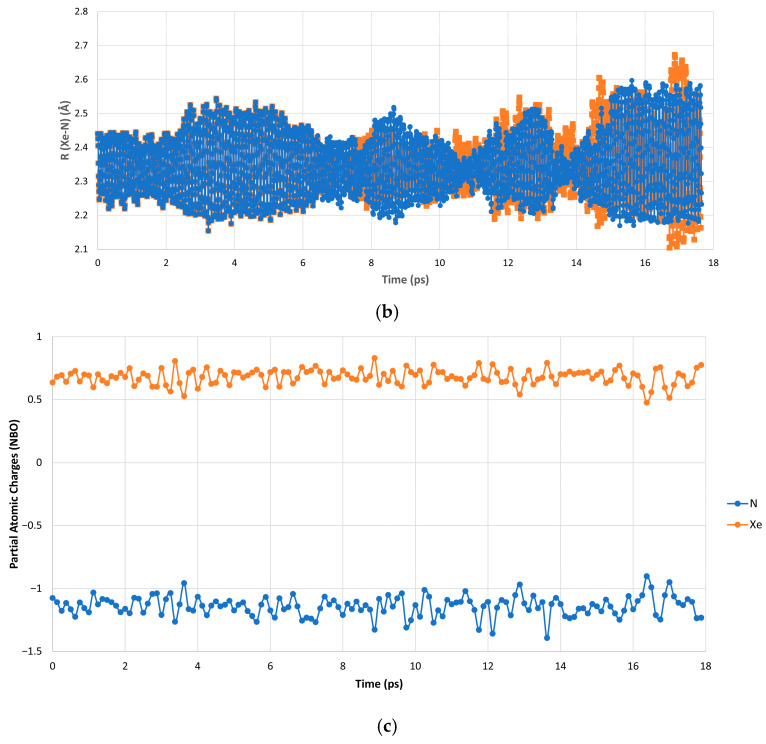
HXeNH_2_ trajectories at 40 K: (**a**) Snapshots along the trajectory. (**b**) The fluctuations of the Xe-N bond length along each trajectory. Each color represents one of the trajectories. (**c**) The NBO partial atomic charges of the Xe and N atoms during a trajectory (H atoms removed for clarity).

As noted above, the noble-gas hydrides are usually prepared at cryogenic temperatures of about 10 K and below. Thus, two trajectories at each temperature of 8 K, 20 K, 40 K, and 60 K for a duration of about 18 ps were performed using AIMD simulations (see Section 3). However, none of these trajectories in any of the temperatures that were checked showed any significant occurrences, such as the dissociation of HXeNH_2_. Figure 3 shows an example trajectory at 40 K.

Not surprisingly, the increase in the simulation temperature from 8 K to 20 K, for example, elevates the structural fluctuations in the molecule—again, without causing the molecule to decompose. Table 1 depicts the quantitated changes in the bond lengths and the H-Xe-N angle between the trajectories, for instance, at 8 K and 20 K.

**Figure 4:**
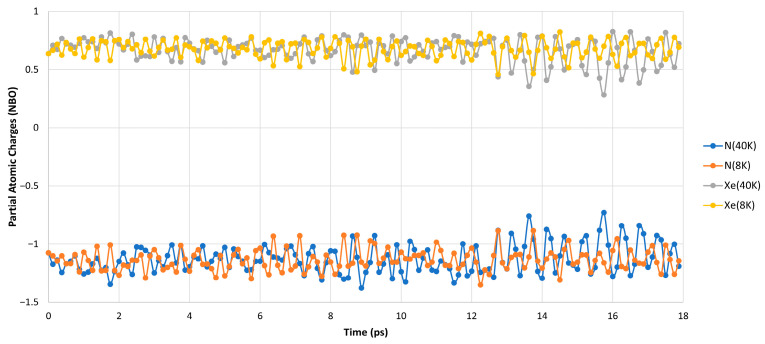
The dynamics of the NBO partial atomic charges of Xe and N atoms along the trajectory at 8 K versus 40 K(H atoms removed for clarity).

As expected, Table 1 shows the increase in the fluctuations of the H-Xe and Xe-N bond lengths and the bending motion of the H-Xe-N angle. This is being reflected by the growing range of their values as the temperature rises. While both bond lengths fluctuate very closely to their CCSD(T) values, the H-Xe-N angle changes further away from its optimized value. This points of course to the higher stiffness of the H-Xe and Xe-N degrees of freedom (DOF) versus the H-Xe-N bending motion. In addition to the increase in the structural fluctuations, the rising temperature also amplifies the fluctuations in the distribution of the partial atomic charges in the molecule. As the atoms move more rapidly due to the growing temperature, so do the modifications to the interactions between them. These of course translate to changes in the electronic density distribution among the atoms. The latter affects directly the results obtained with the NBO method, which depends on the electronic density [43]. The effect of the rising temperature on the fluctuations in the partial atomic charges is shown in Figure 4.

**Figure A1:**
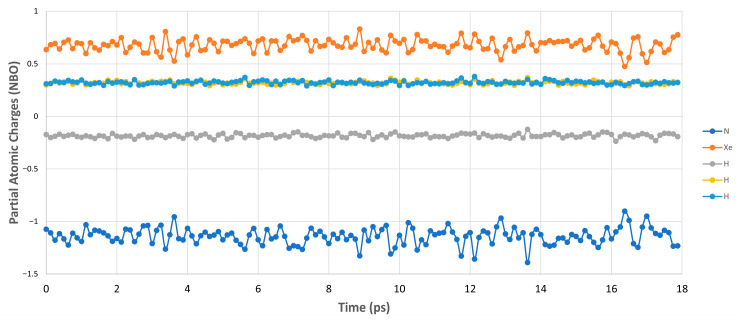
The NBO partial atomic charges along a trajectory at 40 K.

The results show that while the growing temperature amplifies the fluctuations in the partial atomic charges, these occur mostly on the xenon and nitrogen atoms. This is of course understandable as these atoms are much bigger than hydrogen and, thus, have more “electronic density flexibility”. The same behavior can also be seen at each temperature alone (see Figure A1 in Appendix A).

Given the stability of HXeNH_2_ in all trajectories at 8 K, 20 K, 40 K, and 60 K, it is reasonable to assume that given enough trajectories or a longer simulation timescale, the compound would dissociate at least in some of these trajectories, namely, the energy will “flow” and accumulate in the “correct” DOF, which will enable the molecule’s decomposition. Thus, a single trajectory with a longer timescale was applied (see Section 3).

**Figure 5:**
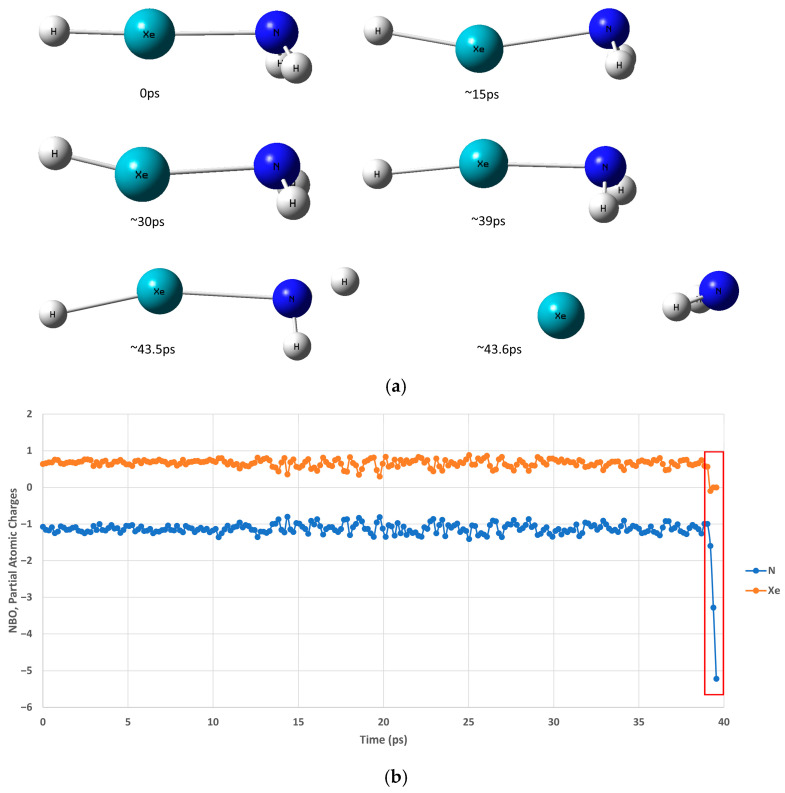
HXeNH_2_ trajectory with a longer timescale at 8 K: (**a**) Snapshots along the simulation taken at various time steps. (**b**) The NBO partial atomic charges of Xe and N atoms along the trajectory (H atom data removed for clarity). The dissociation time region of HXeNH_2_ is emphasized by a red rectangle.

With no exception, all longer timescale trajectories showed the dissociation of HXeNH_2_ into its three constituents, H+Xe+NH_2_. Figure 5 presents these longer timescale trajectories at 8 K.

The results show that after about 43.5 ps, HXeNH_2_ starts to dissociate into its three constituents, H+Xe+NH_2_, as can be seen from the snapshots taken from the trajectory (Figure 5a). In addition, plotting the H-Xe and Xe-N bond lengths and H-Xe-N angle along the trajectory shows that the bonds are elongated until they eventually break, while the H-Xe-N angle keeps fluctuating between 155 and 177 degrees.

Most interestingly, the dissociation of HXeNH_2_ occurs due to an abrupt change in the electronic density distribution after only about 39 ps, as can be seen in Figure 5b. The analysis of the trajectory exhibits the following dissociation mechanism for HXeNH_2_: First, a charge–transfer step occurs after about 39 ps. This is followed by the breaking down of the H-Xe bond, which is followed almost immediately by the decomposition of the X-Ne bond, at ~43.5 ps. In addition, and most importantly, this mechanism occurred in all cases where HXeNH_2_ dissociated. It should be noted that this result corresponds well with the known lower barrier for this dissociation pathway than that which decomposes HNgY into the Ng + HY constituents.

It should be noted that the results were obtained with MP2, which is a single-reference method. Obviously, such a single-reference framework cannot be expected to describe adequately the multi-reference dissociation and its products, H + Xe + NH_2_, since the products include open-shell species. However, the MP2 procedure, much like other single-reference techniques, should be valid up to the charge transfer decomposition region. In addition, as the trajectories reach this region, the system’s geometry indicates that the products should be H + Xe + NH_2_. Thus, as a single-reference method, the MP2 framework can take us almost to the point of decomposition and tell us what the products should be. Furthermore, the “static” multi-reference MRCI calculation shown above indicates that in this region, the species formed, corresponding to the three constituents of H + Xe + NH_2_. Thus, this higher-level calculation supports the decomposition mechanism and its products. Furthermore, where valid, the MP2 method can describe changes in the electronic distribution, as reflected by the changes in the partial atomic charges along the trajectory which leads to dissociation.

In general, the stability of the noble-gas hydrides at cryogenic temperatures is an experimental fact. As noted earlier, the calculated barrier for the similar compound HArF was just 0.18 eV in vacuum. However, the compound was found experimentally while prepared in a rare-gas matrix. As for the relevance of tunneling to this study, all the trajectories were carried out at energies well above the computed barrier (e.g., kinetic energy of ~0.43 and above in the cases shown). Thus, tunneling contributions to the dynamic simulations presented here should be negligible. Therefore, it does not seem that tunneling calculations are necessary for this study.

At all temperatures and trajectory timescales, the H-Xe and Xe-N bonds show, as expected, a frequency-like behavior which is more pronounced for the Xe-N bond (Figure 3b shown for the Xe-N bond). This is due to the faster vibration of the H-Xe bond and its modification by its relatively large and rapid rotational movement.

The apparent strange behavior of the partial atomic charges around 39 ps deserves an explanation (Figure 5b, enclosed by a red rectangle). The figure shows that while the partial charge of the Xe atom reduces to zero, all other atoms (shown only for N atom, for clarity) become highly negatively charged. However, the trajectories were carried out by the single-reference MP2 method. The redistribution of the electronic density and the introduction of new electronic configurations cannot be adequately described by this single-reference method. The behavior of the partial atomic charge on Xe seem reasonable, as it starts to return to its closed shell. However, the negative partial charges on all other atoms are most probably an artifact due to the failure of the single-reference MP2 method to describe this scenario.

## 3. Computational Methods

The structure of HXeNH_2_ was optimized from a single structure (similar to that in Figure 1) by searching a minimum on the electronic potential energy surface and calculating its vibrational harmonic frequencies at the optimized geometry. This was performed at the MP2 and the CCSD(T) level of theory via the GAUSSIAN16 suite, Revision C.02 [44]. Since the CCSD(T) method requires numerical derivatives, the optimization was carried out using the Eigenvalue-Following algorithm (EF) using a default tight SCF convergence (Tight) and the very tight threshold criterion for the geometrical convergence (10^−6^, VTight). In addition, the need for numerical derivatives necessitated the use of an integration grid of adequately sized grid points (SuperFineGrid).

In order to describe the compound, a mixed basis-set was used, which included the LaJohn Effective Core Potential (ECP) for Xe and the Pople Gaussian basis-set of 6-311++G(2d,2p) for the nitrogen and hydrogen atoms. The LaJohn-18 ECP (LJ-18) consists of 18 explicit electrons and 36 electrons for the ECP region. Although this ECP is not the largest one possible for describing Xe, it should be sufficient together with the above Pople basis-set to describe HXeNH_2_. We note that the mixed basis-set is relatively balanced and has a low Basis-set Super Position Error (BSSE). In addition, and above all, this mixed basis-set and, in particular, the LaJohn-18 ECP, was used successfully in previous studies to predict experimentally found compounds such as HXeCCH [9,45], HXeOH [11,12], and other molecules [24].

The partial atomic charges were calculated at the optimized geometry via the Natural Bond Orbital (NBO) method as applied in the GAUSSIAN16 package [46].

All thermodynamic calculations were carried out at the CCSD(T) level of theory using the same basis-sets stated above, while also taking into account the Zero Point Energy correction (ZPE).

**Figure A2:**
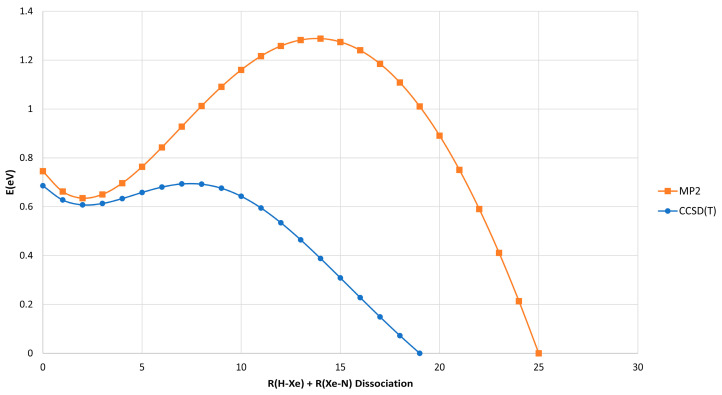
The decomposition pathway of HXeNH_2_ into H+Xe+NH_2_, calculated with CCSD(T) and MP2 methods.

The dissociation of HXeNH_2_ into its three constituents (H+Xe+NH_2_) entails the opening of the closed electronic shell of HXeNH_2_ and, therefore, the need for a multi-reference method. Thus, this calculation was performed at the MRCI level of theory with an active space of 16 electrons and 12 orbitals, using the GAMESS package (2024 R1) [47]. The calculation was performed by extending the H-Xe and the Xe-N bonds by 0.1 Angstrom and conducting a single-point energy calculation at each point along this path. In addition, the same path was also calculated by MP2 and CCSD(T) for comparison (see Figure A2).

Ab Initio Molecular Dynamics (AIMD) simulations for HXeNH_2_ were computed at temperatures of 8 K, 20 K, 40 K, and 60 K. These were simulated using the Born–Oppenheimer Molecular Dynamics method (BOMD) as implemented in the GAUSSIAN16 suite [44]. The trajectories utilized the full MP2 level of theory and using the same basis-sets noted above. The MP2 method was used by us as it was preferrable to DFT, since in MP2, the long-range interactions that play a significant role here are included as a first principle intrinsic to this method. The initial conditions for these trajectories were controlled by choosing the initial rotational energy from a thermal distribution, assuming a symmetric top (RTemp option). At first, two trajectories at each temperature were computed for 12,000 steps (~18 ps). Next, a single longer trajectory was calculated for 100,000 steps (~150 ps) at 8 k and 20 K, and for 36,000 steps (~54 ps) at 40 K. The analysis of these trajectories was performed using every 10th snapshot for the longer trajectories at 8 K and 20 K and every 4th snapshot for all other trajectories (see Table A2 in Appendix A).

Finally, the analysis of the fluctuations in the partial atomic charges along the trajectories was achieved by extracting the coordinates of 144 (every 125 fs) and 250 (every 175 fs) snapshots at 8 K for the relatively short and longer trajectories accordingly. In addition, 250 snapshots were extracted from the longer trajectory at 20 K. Additionally, 144 and 216 sets of coordinates were extracted from the short and longer trajectories at 40 K, respectively (see Table A2 in Appendix A). The extracted coordinates were then used as the basis for a single-point (SP) energy calculation together with a computation of the NBO charges at each snapshot taken. Both the extraction and the preparation of the input files for calculating the NBO charges at each snapshot were facilitated by python scripts developed for this purpose (the scripts are available by email request).

## 4. Conclusions

Here, we studied the stability and decomposition dynamics of the recently predicted noble-gas hydride compound type, HXeNH_2_. This was carried out using ab initio calculations and AIMD simulations. The understanding of the dynamics of the molecule’s decomposition and, in particular, its dissociation mechanism may help in the experimental realization and understanding of this compound and perhaps other Xe-N bond-containing compounds.

The results imply that at all temperatures checked, the compound dissociates into its three constituents, H+Xe+NH_2_. Most importantly, the study discovered a distinct mechanism for the molecule dissociation which is seen for all temperatures checked. A key ingredient in this mechanism is a charge–transfer step which precedes the structural modifications. The latter steps include the H-Xe bond breaking, which is followed almost immediately by the dissociation of the slower moving Xe-N bond.

It should be noted that in its validity domain, the MP2 method can describe changes in the electronic state, as reflected by the changes in the partial charges of the atoms in the system, along the trajectory. This, of course, cannot be said of simulations based on a classical force field type.

Finally, we also pointed out as a speculation, the possible connection between HXeNH_2_ and the known and long-standing puzzle of the “missing” Xe phenomenon in the Earth’s atmosphere. Specifically, we speculate that Xe may be contained as HXeNH_2_ in the crust of the Earth.

We hope that this study stimulates more research on the decomposition dynamics and the dissociation mechanism of this intriguing and rather unique type of molecules and perhaps even their possible connection to other areas of science.

## Figures and Tables

**Table 1:** The table presents the H-Xe and Xe-N bond lengths together with the H-Xe-N angle, alongside their corresponding optimized CCSD(T) value (left most column). The minimum, maximum, and averaged values in these degrees of freedom at 8 K and 20 K are also shown ([min, max, Av.]). Bond lengths are in Angstrom and angles in degrees.

	8 K [Min, Max, Av.] (Å/Degrees)	20 K [Min, Max, Av.] (Å/Degrees)
R (H-Xe, 1.84 Å)	[1.59, 2.15, 1.83]	[1.47, 2.21, 1.84]
R (Xe-N, 2.35 Å)	[2.10, 2.71, 2.36]	[2.08, 2.73, 2.36]
A (H-Xe-N, 177.5°)	[156.5, 179.8, 170.7]	[155.1, 179.9, 171.3]

## Data Availability

Python scripts are available on request by email from the authors.

## Abbreviations

The following abbreviations are used in this manuscript:

Ng	Noble gas
AIMD	Ab Initio Molecular Dynamics
BOMD	Born–Oppenheimer Molecular Dynamics
DOF	Degrees of Freedom
NBO	Natural Bond Orbital
ECP	Effective Core Potential
CCSD(T)	Coupled Cluster of Singles Doubles and perturbative Triples
MRCI	Multi-reference Configuration Interaction
ZPE	Zero-Point Energy
LJ-18	LaJohn-18 ECP basis-set
MP2	Møller–Plesset 2nd order perturbation theory

## Appendix A

We provide here additional data on the structure, properties, and some technical data regarding the AIMD trajectories of HXeNH_2_ which were not included in the main text. Specifically, the additional information includes the full span of the harmonic vibrational analysis of the compound, the optimized coordinates of HXeNH_2_, and details of the runs and analysis of the AIMD trajectories. In addition, a comparison for the HXeNH_2_ dissociation path to H+Xe+NH_2_ at the MP2 and CCSD(T) level of theory is provided.

**Table A1:** Full vibrational mode analysis of HXeNH_2_.

Mode	CCSD(T) Freq. (cm^−1^)	Mode Description
1	324.04	Xe-NH_2_ Str.
2	515.76	Bend
3	537.80	Bend
4	785.82	Bend
5	821.70	H + NH_2_ Symm. bend
6	1318.50	H-Xe Str. + NH_2_ Symm. bend
7	1580.88	H-Xe str. + NH_2_ Symm. bend
8	3421.97	NH_2_ Symm. Str.
9	3509.04	NH_2_ Asymm. Str.

**Table A2:** AIMD running and analysis details.

T	Number of Trajectories	Duration	General Analysis Every n^th^ Snapshot	Number of Calculated NBO Snapshots (Every Number fs)
8 K	2	~18 ps	4	144 (125 fs)
	1	~150 ps	10	250 (175 fs)
20 K	2	~18 ps	4	
	1	~150 ps	10	250 (77 fs)
40 K	2	~18 ps	4	144 (125 fs)
	1	~54 ps	4	216 (125 fs)
60 K	2	~18 ps	4	

**Table A3:** HXeNH2: Optimized CCSD(T) cartesian coordinates (in Angstrom).

Atom	X	Y	Z
N	0.000000	0.000000	0.000000
Xe	0.000000	0.000000	2.349089
H	0.080033	0.000000	4.191635
H	−0.618583	0.777530	−0.228302
H	−0.556748	−0.823225	−0.227285
